# Incorporating Drought and Submergence Tolerance QTL in Rice (*Oryza sativa* L.)—The Effects under Reproductive Stage Drought and Vegetative Stage Submergence Stresses

**DOI:** 10.3390/plants10020225

**Published:** 2021-01-24

**Authors:** Asmuni Mohd Ikmal, Abd Aziz Shamsudin Noraziyah, Ratnam Wickneswari

**Affiliations:** Department of Biological Sciences and Biotechnology, Faculty of Science and Technology, Universiti Kebangsaan Malaysia, Bangi 43600, Selangor, Malaysia; mohdikmal@siswa.ukm.edu.my

**Keywords:** drought, submergence, *Sub1*, *qDTY*, rice, marker-assisted selection, morpho-physiology, agronomy

## Abstract

Drought and submergence have been the major constraint in rice production. The present study was conducted to develop high-yielding rice lines with tolerance to drought and submergence by introgressing *Sub1* into a rice line with drought yield QTL (*qDTY*; *QTL* = quantitative trait loci) viz. *qDTY_3_._1_* and *qDTY_12_._1_* using marker-assisted breeding. We report here the effect of different combinations of *Sub1* and *qDTY* on morpho-physiological, agronomical traits and yield under reproductive stage drought stress (RS) and non-stress (NS) conditions. Lines with outstanding performance in RS and NS trials were also evaluated in vegetative stage submergence stress (VS) trial to assess the tolerance level. The QTL class analysis revealed *Sub1* + *qDTY_3_._1_* as the best QTL combination affecting the measured traits in RS trial followed by *Sub1* + *qDTY_12_._1_*. The effects of single *Sub1*, *qDTY_3_._1_* and *qDTY_12_._1_* were not as superior as when the QTLs are combined, suggesting the positive interaction of *Sub1* and *qDTY*. Best performing lines selected from the RS and NS trials recorded yield advantage up to 4453.69 kg ha^−1^ and 6954 kg ha^−1^ over the parents, respectively. The lines were also found having great tolerance to submergence ranging from 80% to 100%, contributed by a lower percentage of shoot elongation and reduction of chlorophyll content after 14 days of VS. These lines could provide yield sustainability to farmers in regions impacted with drought and submergence while serving as important genetic materials for future breeding programs.

## 1. Introduction

Over the past years, farmers have been cultivating high-yielding rice cultivars in their field to obtain higher income and meet the demand for rice. However, heightening climate change has caused more incidents of drought and flood around the world and is retarding rice production. Drought has been the key concern for food security and caused $28 billion in losses to the crop and livestock industry in Asia from 2003 to 2013 [1]. Farmers in South-East Asian countries especially Laos, Thailand, Malaysia, Philippines and Indonesia were finding it difficult to plant rice in their fields due to water shortages throughout 2019 to early 2020 [2,3,4]. According to Jusop [4], farmers in northern peninsular Malaysia were more worried about the drought that destroyed their rice field than the coronavirus disease 2019 (COVID-19) pandemic. Moreover, faulty irrigation equipment in the fields has worsened the condition of drought in 1000 hectares of paddy field in Kepala Batas [5].

Just as rice cultivation can be affected by drought, floods also cause severe damage to rice. Almost 40 million hectares of rice field around the world were damaged by flooding [6,7]. It is estimated that 4 million tonnes of rice were lost due to floods in India, where 30% of the rice field is prone to flooding [8,9]. The effect of the flood to Malaysian farmers caused around RM2 million in losses [10,11]. Malaysia is most likely to be affected by drought and floods that are caused by prolonged dry phases and a substantial amount of rainfall during the monsoon season. The main rice cultivating areas are equipped with an irrigation system, but farmers outside these areas are facing difficulties in watering their fields as they rely solely on rainfall. The current Malaysian high-yielding rice cultivars planted by farmers are susceptible to drought and submergence and research on the development of drought and submergence tolerant cultivars started just a few years ago.

Marker-assisted quantitative trait loci (QTL) pyramiding was conducted before to develop rice lines in a popular mega-variety of Malaysia, MR219 with the drought yield QTL (*qDTY*) which provides considerable tolerance to drought [12]. The lines were advanced and tested in several locations around Malaysia. However, the lines had low to moderate tolerance to submergence as evaluated by Ikmal et al. [13]. *Sub1* which provides tolerance of 2–3 weeks of submergence was extensively used in multiple breeding programs to develop submergence tolerant cultivars in South-East Asian countries such as the Philippines, India and Indonesia [14,15] since its discovery by Xu and Mackill [16]. The cultivar FR13A was identified as the donor of the *Sub1* and described as the best source of submergence tolerance.

In previous studies, some prominent drought-tolerant varieties such as Nagina 22, Apo, Kali Aus, Dular, Kalia, and Adey Sel were used in conventional breeding programmes [17]. QTL validation undertaken on a large set of drought-tolerant genotypes found that the major-effect QTL *qDTY_12_._1_* was present in 85% of these lines whereby 50% of the lines contained the drought grain yield QTLs*, qDTY_1_._1_, qDTY_1_._2_, qDTY_2_._1_, qDTY_3_._1_, qDTY_3_._2_* and *qDTY_8_._1_* [18]. Two major effect QTLs, namely *qDTY_3_._1_* and *qDTY_12_._1_*, were reported in a population of Apo/2*Swarna [19] while *qDTY_3_._1_* was also identified by Dixit et al. [20]. Many of the *qDTY*s found were introgressed and pyramided into high-yielding but drought-susceptible varieties around the world, including Anjali, Kalinga, IR64, TDK1, Swarna, Sabitri, and Jinmibyeo [17]. These improved varieties have proven to give a yield advantage and survived well under drought. However, the identified *qDTY*s generally have a genetic gain of 10% to 30%, with a yield advantage of 150 to 500 kg ha^−1^ over recipient parents [21]. According to Swamy and Kumar [18], to provide a significant economic impact for rice farmers, at least 1.0 t/ha of yield advantage needs to be achieved. Therefore, the introgression of only one QTL for drought tolerance will unlikely give better results for breeding drought-tolerant cultivars.

Studies have proven the effectiveness of the *Sub1* for tolerance to submergence when introgressed into their popular but submergence-susceptible cultivars. Development of drought and submergence rice cultivars is given serious attention by many researchers around the world to mitigate their disastrous effects. The rice cultivars could provide better yield insurance in countries facing both or either one of the natural disasters. The present study used a combined strategy of genotyping at the seedling stage and phenotyping at reproductive and maturity stage for selection of genotypes with the desired QTLs and good phenotypic traits.

Recent studies have demonstrated the benefits of combining the QTL for tolerance to abiotic stresses [14,22,23]. However, their studies used different populations and different QTLs which would have a different effect on different genetic background and were only limited to days to flowering (DTF), plant height (PH) and grain yield (GY). Therefore, this study was conducted with the objectives to develop drought and submergence tolerant rice lines with the background of UKM5 harbouring *qDTY_3_._1_* and *qDTY_12_._1_* and IR64-*Sub1* harbouring the tolerant *SUB1*, and to evaluate the effects of distinct combinations of *Sub1* and *qDTY* on morpho-physiological, agronomical and yield, and the performance of breeding lines with different QTL combinations under reproductive stage drought stress (RS), non-stress (NS) and vegetative stage submergence (VS) conditions.

## 2. Results

### 2.1. Development of Lines Using Marker-Assisted Breeding

A total of 143 putative F_1_ seeds were obtained after 21 days of crossing. Confirmation using polymorphic markers showed 72% of the seeds were hybrid. Twenty-five F_1_ individual plants with good phenotypes were chosen for backcrossing. From 470 BC_1_F_1_ individuals generated, 175 individuals with desirable PH, number of panicles (NP) and DTF between 90–110 days were selected. Selected BC_1_F_1_ individuals were advanced to BC_1_F_2_ generation. A total of 326 individuals were selected from 1750 BC_1_F_2_ individuals planted. Individuals with homozygous alleles for the target loci were forwarded to BC_1_F_3_ generation.

### 2.2. Imposition of Drought

Water depth level and rainfall data are presented in Figure S1. RS was initiated on 1 June 2019 and on 18 June 2019, the water depth dropped to 10 cm below ground level. However, due to several days of rain, the water depth increased. The lowest water depth recorded was 80 cm below ground level. Starting from the end of June, most genotypes started booting and began to flower. The imposition of drought at this stage is critical to discriminate genotypes with good tolerance to drought. Even though there were several rainy days during stress imposition, water was drained immediately. In this condition, drought can be considered to occur at multiple times with one to two days or rainy days interval. The lowest minimum daily temperature recorded was 22 °C while the highest maximum daily temperature was 33 °C.

### 2.3. Performance of Lines under Reproductive Stage Drought Stress (RS)

Table 1 shows the range, mean ± standard error of the mean (SE), coefficient of variation (CV) and trial broad-sense heritability (*H*). Five traits namely DTF, PH, chlorophyll content (CC), panicle length (PL) and thousand-grain weight (TGW) recorded low CV (<10%) in NS and RS trials. Number of spikelets per panicle (SPP) and spikelet fertility percentage (SFP) recorded medium CV (10–20%), while NP, number of filled spikelets per panicle (FS) and GY recorded high CV (20–30%) in the NS trial. In RS trial, NP had high CV (20–30%) while GY had very high CV (>30%). All traits had moderate to high *H* in NS trial except NP which was low (0.30). DTF, CC and GY recorded very high *H*. In RS trial, *H* of GY was higher (0.97) compared to in NS trial (0.93). Other traits also had higher *H* in RS trial compared to in NS trial except CC (NS = 1.00, RS = 0.98) and SFP (NS = 0.75, RS = 0.56).

Results of the analysis of variance for all traits in NS and RS trials and the mean values for all traits recorded for BC_1_F_4_ lines and checks are presented in Table S1, Table S2, Table S3 and Table S4, respectively. All BC_1_F_4_ lines showed DTF between 68 to 81 days in NS trial and between 85 to 101 days in RS trial. In RS trial, MR219 took 30 days longer to reach DTF compared to the NS trial. Drought-tolerant checks used in this study, NMR152 had an earlier DTF in NS trial (82 days) compared to in RS trial (83 days). The other QTL donors; IR64-*Sub1*, IR81896 and IR84984 also had a delayed DTF in RS trial compared to in NS trial.

The majority of the genotypes tested had reduced PH in the RS trial compared to in the NS trial except for GEN124 and GEN170. GEN170 had the lowest PH in the NS trial (68.00 cm) while other BC_1_F_4_ lines and checks had PH between 86.50 to 120.50 cm. GEN260 and GEN170 had the tallest PH among BC_1_F_4_ lines in the NS trial (115.00 cm) and RS trial (103.00 cm), respectively. The PH of MR219 reduced by 12.87% from 101.00 cm to 88.00 cm in the RS trial while the tolerant check, NMR152 reduced by 16.07% from 112.00 cm to 94.00 cm. UKM5 showed a greater reduction of PH (21.86%) compared to MR219 and NMR152.

All BC_1_F_4_ lines had reduced NP in the RS trial compared to the NS trial except GEN117, GEN160, GEN220, GEN228 and GEN264. The highest NP (25.00) in the NS trial was recorded for GEN269, GEN230 and GEN124 while GEN264 recorded the highest NP (22.00) in the RS trial. Meanwhile, GEN215 produced the lowest NP in the NS trials. NMR152, IR81896 and IR84984 produced NP between 15.50 to 21.50 in the NS trial and between 5.50 to 13.50 in the RS trial. NP of MR219 was reduced by 61.75% in the RS trial compared to the NS trial while UKM5′s NP was reduced by 37.21% in the RS trial compared to the NS trial. NP of IR64-*Sub1* was reduced by 22.58% from 15.50 in the NS trial to 12.00 in the RS trial.

Chlorophyll content (CC) of BC_1_F_4_ lines was reduced in the RS trial compared to NS trial except for GEN227, GEN240, GEN234 and GEN266. The highest CC in the NS trial was recorded for GEN207 (50.00) while the lowest was recorded for GEN250 (31.50). The highest CC in the RS trial was recorded for GEN234 (47.10) while the lowest was recorded for NMR152 (18.30). GEN188 had the longest PL (29.50 cm) in the NS trial followed by GEN216 (28.80 cm), GEN128 (28.00 cm), GEN147 (28.00 cm) and GEN191 (27.55 cm). These BC_1_F_4_ lines had longer PL than all the checks. Among the checks, MR219 and NMR152 had the shortest PL. Meanwhile, UKM5 had longer PL than IR64-*Sub1*. In the RS trial, GEN148 had the longest PL of at 31.15 cm followed by GEN249 (28.80 cm) and it was longer than all checks.

In the NS trial, GEN188 had the highest number of SPP (190.50) followed by GEN116 (184.00) and GEN187 (183.50). The lowest SPP was recorded for GEN117 (73.00). GEN188′s SPP was higher than all checks, including UKM5 (147.00). Meanwhile, the highest SPP in the RS trial was recorded for GEN148 (196.50). GEN263 recorded the lowest SPP in the RS trial (74.00). MR219, NMR152 and IR81896 had higher SPP in the RS trial compared to the NS trial. Some BC_1_F_4_ lines also had higher SPP in the RS trial, such as GEN112 and GEN117.

GEN116, which had high SPP recorded the highest FS in the NS trial (158.50). GEN193 had the lowest FS in NS trial. Among the checks, UKM5 had the highest FS (111.50) while the lowest was NMR152 (76.50). In RS trial, GEN148 had the highest FS compared to other BC_1_F_4_ lines and checks. IR64-*Sub1* had the lowest FS compared to other checks.

GEN170 had the highest percentage of SFP (98.07%) followed by GEN266 (97.55%). However, the SFP dropped to 75.83 and 86.44%, respectively in the RS trial. The highest SFP in the RS trial was recorded by GEN263 (91.96%) while the lowest was recorded by GEN229 (39.06%). All SFP of checks were lower in the RS trial compared to the NS trial. GEN124 recorded the highest TGW in the NS trial (27.70 g), while the lowest was recorded for GEN187 (22.80 g). In the RS trial, GEN267 recorded the highest TGW (26.85 g) compared to other BC_1_F_4_ lines and checks. GEN124 recorded the lowest TGW (17.40 g) in the RS trial.

Most BC_1_F_4_ lines had GY more than 6000 kg ha^−1^ in the NS trial. From the overall 85 BC_1_F_4_ lines evaluated, 17 lines had GY more than 10,000 kg ha^−1^. GEN206 recorded the highest GY in the NS trial (13892.80 kg ha^−1^), while the lowest was recorded for GEN215 (3616.54 kg ha^−1^). In the RS trial, the highest GY was recorded for GEN239 (4796.35 kg ha^−1^) compared to other BC_1_F_4_ lines and checks. GEN146 had the lowest GY among BC_1_F_4_ lines but was slightly higher than IR64-*Sub1*.

### 2.4. Effects of Different Combinations of Quantitative Trait Loci (QTLs) on Measured Traits

All 85 BC_1_F_4_ lines selected for evaluation in the NS and RS trials were assigned to their respective QTL classes based on the genotypic data obtained after polymerase chain reaction (PCR). Among the selected BC_1_F_4_ lines, no line was classified into class D (*qDTY_3_._1_* + *qDTY_12_._1_*). Significant differences were found between QTL classes and checks for all traits in both NS and RS except for NP and GY in NS and SFP in RS (Table 2 and Table 3). All QTL classes were only differed by one to two days of DTF in NS trial. In the RS trial, more variation can be observed between QTL classes. Class F had the earliest DTF (75 days) than the others while Class A and Class G took the longest DTF. All checks flowered earlier (84 to 85 days) than BC_1_F_4_ lines except for MR219.

All QTL classes also experienced a reduction of PH in the RS trial compared to the NS trial. Even though detected as significantly different from each other, the means for the PH of QTL classes were almost similar and differed only by 2.00 cm except for Class A. Class A had the highest NP in the RS trial compared to other QTL classes and was superior to IR81896, IR84984 and MR219. However, Class A did not produce the highest NP in the NS trial and was outperformed by Class C, E and F which also produced higher NP than MR219, IR64-*Sub1*, IR81896 and IR84984. Class A had the highest CC in the RS trial compared to other QTL classes and all checks. Class G recorded the lowest CC compared to other classes but higher than IR64-*Sub1*, NMR152, IR84984 and UKM5. In NS trial, all QTL classes recorded CC of more than 40.00 and were higher than MR219, IR64-*Sub1* and NMR152.

Moreover, Class C had the longest PL (25.81 cm) while Class G had the shortest PL (23.37 cm). As compared to Class C, all checks had shorter PL except IR84984 (26.35 cm). In the RS trial, Classes A, B and G were the classes that had longer PL compared to the NS trial. IR64-*Sub1*, MR219 and IR81896 also had longer PL in the RS trial compared to the NS trial. Three classes (B, E and G) had higher SPP in the RS trial compared to the NS trial. The susceptible check, MR219 also had higher SPP in the RS trial. Among the classes, Class C (130.96) and Class B (151.67) had the highest SPP in the NS and RS trials, respectively. Class F had the highest FS in the NS trial while Class B had the highest FS in the RS trial. The FS of Class F in the NS trial was higher than all checks except UKM5 while FS of Class B in the RS trial was higher than all checks except MR219 and IR81896. The highest SFP in the NS trial was recorded for Class F but lower than MR219, IR64-*Sub1* and IR81896. The same situation also can be observed in the RS trial where Class F had the highest SFP among other classes but was lower than IR64-*Sub1* and MR219.

For TGW, Class A topped the other classes in the NS trial and was higher than all checks (Table 3). However, in the RS trial, Class B recorded the highest TGW than the other classes and was higher than IR64-*Sub1* and IR81896. GY of class C was the highest compared to other classes in the NS trial but was lower than NMR152 and UKM5. Even though Class C recorded the highest GY in the NS trial, its GY in the RS trial was lower than Class B, NMR152, IR81896 and UKM5. Class B had the highest GY (2427.45 kg ha^−1^) in the RS trial while the lowest GY among the classes was recorded by Class A (769.22 kg ha^−1^).

### 2.5. Correlation

**Figure 1:**
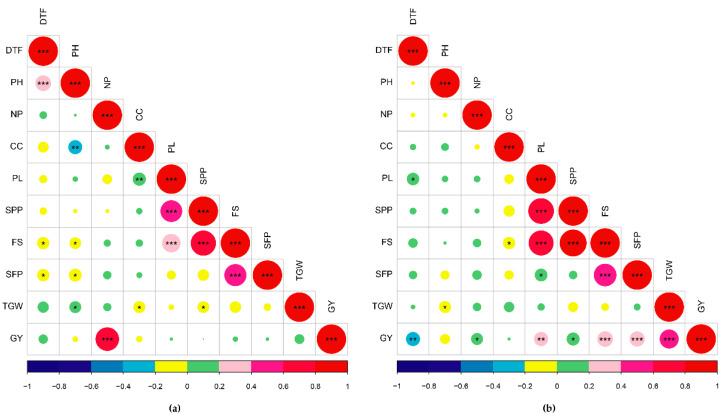
Graphical correlation matrix for traits evaluated in (**a**) non-stress and (**b**) reproductive stage drought stress trials in 2019MS. DTF, Days to flowering; PH, plant height; NP, number of panicles; CC, chlorophyll content; PL, panicle length; SPP, number of spikelet per panicle; FS, number of filled spikelet per panicle; SFP, spikelet fertility percentage; TGW, thousand-grain weight; GY, grain yield; * significant at *p* < 0.05, ** significant at *p* < 0.01, *** significant at *p* < 0.001.

Figure 1a,b shows the graphical correlation matrix for the traits evaluated in the NS and RS trials respectively. In the NS trial, GY showed significant high positive correlation with NP (*r* = 0.80, *p* < 0.001). The yield related traits; PL, SPP and FS were positively correlated with GY but was low. TGW was significantly positively correlated with PH (*r* = 0.21, *p* < 0.05). PL was significantly positively correlated with CC (*r* = 0.22, *p* < 0.05), SPP (*r* = 0.55, *p* < 0.001) and FS (*r* = 0.39, *p* < 0.001) but was negatively correlated with SFP (*r* = −0.11, *p* > 0.05).

In the RS trial, GY was significantly positively correlated with NP (*r* = 0.16, *p* < 0.05), PL (*r* = 0.22, *p* < 0.01), SPP (*r* = 0.19, *p* < 0.05), FS (*r* = 0.27, *p* < 0.001), SFP (*r* = 0.25, *p* < 0.001) and TGW (*r* = 0.38, *p* < 0.001) while negatively correlated with DTF (*r* = −0.22, *p* < 0.01). PL was found to have positive correlation with SPP (*r* = 0.75, *p* < 0.001), FS (*r* = 0.72, *p* < 0.001) and SFP (*r* = 0.18, *p* < 0.05) and TGW (*r* = 0.08, *p* > 0.05). NP had positive correlation with PL, SPP, FS and SFP but it was low and non-significant.

### 2.6. Selection of Superior Lines Using Multivariate Analysis

**Figure 2:**
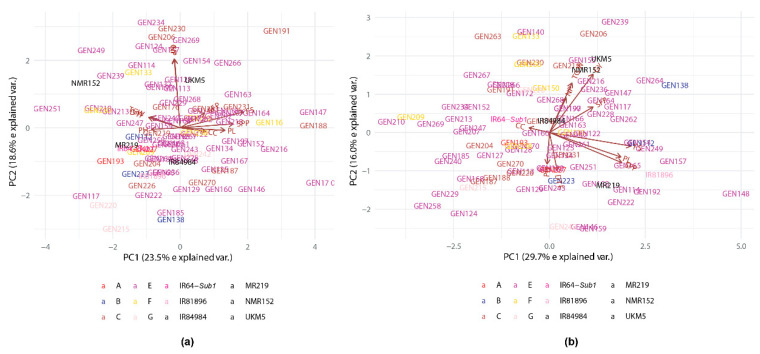
Principal component analysis biplot for 10 traits and 91 genotypes evaluated in (**a**) non-stress and (**b**) reproductive stage drought stress trials in 2019MS.

The first PC (PC1) of the principal component analysis (PCA) which is usually the largest, explained 23.46% of the total variations, while PC2 accounted for 18.57% of the total variations, which summed up to 42.03% for NS trial (Figure 2a). The PCA variable loadings indicated that FS (0.559) and SPP (0.469) are positively associated with PC1 and gave the largest contributions (Table S5). For PC2, the largest contributing variable to the total variation explained was NP (0.683) and GY (0.679). Genotypes from Class C and E were scattered in the upper quadrant of the biplot, which indicates a higher GY. Genotypes from Class G which had the lowest GY in the NS trial were scattered in the lower quadrant of the biplot.

**Figure 3:**
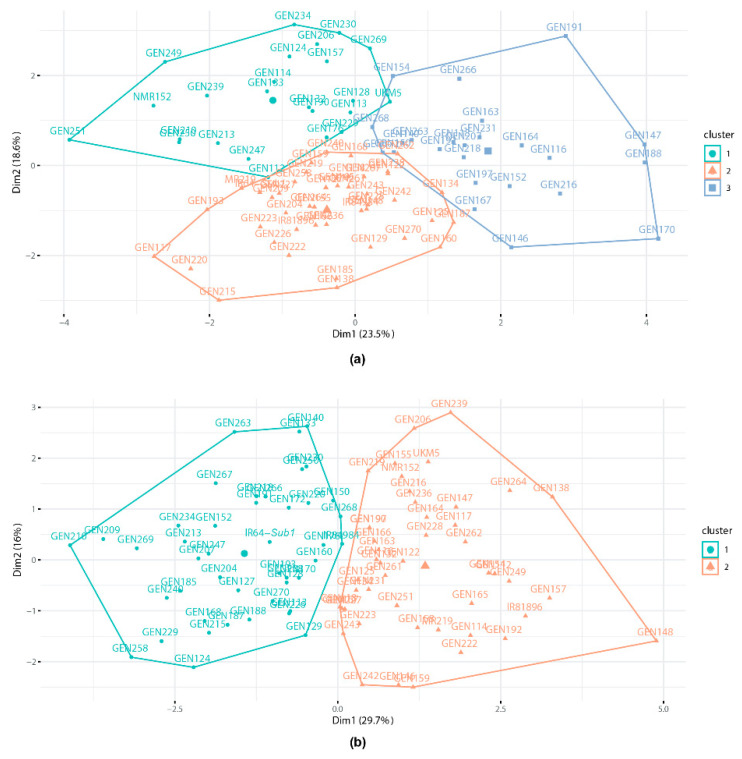
Cluster plot showing the clustering of genotypes in (**a**) non-stress and (**b**) reproductive stage drought stress trials in 2019MS.

To obtain more information on the discrimination of genotypes, clustering using the K-means method was undertaken. The optimal number of clusters was determined using the silhouette method and genotypes were grouped into three clusters (Figure 3a). Based on the cluster means shown in Table S6, Cluster 1 was occupied by NMR152, UKM5 and BC_1_F_4_ lines with high DTF, NP, TGW and GY such as GEN112, GEN133, GEN206, GEN230 and GEN239. Cluster 2 was occupied by most genotypes with lower GY including IR64-*Sub1*, IR81896, IR84984 and MR219 while Cluster 3 was occupied by BC_1_F_4_ lines with high CC, PL, SPP, FS and SFP such as GEN140, GEN147 and GEN263. However, GY of the lines were lower than genotypes in Cluster 1.

Figure 2b shows the biplot of the principal component analysis (PCA) performed using all ten traits in RS trial. The total variations explained were 29.74% and 16.04% for PC1 and PC2, respectively. The PCA variable loadings indicated that PC1 was largely contributed and positively associated with PL (0.481), SPP (0.487) and FS (0.549). PC2 was largely contributed and positively associated with NP (0.318), GY (0.535) and TGW (0.455) (Table S7). BC_1_F_4_ lines categorized in Classes A, B and G were scattered in upper quadrants of the biplot which indicates lower GY than lines scattered at the lower quadrants. GEN239 and GEN206 had positive PC1 scores with very high positive PC2 scores, which indicate good PL, SPP, FS and higher GY than other genotypes. IR64-*Sub1* and IR81896 had low positive PC2 scores and negative PC1 scores, which indicates lower GY, PL, SPP and FS.

The optimal number of clusters determined using the silhouette method and genotypes were grouped into two clusters using the K-means method (Figure 3b). Based on the cluster means in Table S6, Cluster 2 was occupied by genotypes with higher NP, PL, SPP, FS, SFP, TGW and GY than genotypes in Cluster 1. BC_1_F_4_ lines grouped in Cluster 2 include GEN112, GEN147, GEN155, GEN206, GEN219 and GEN239. As in the NS trial, the drought-tolerant checks; UKM5, IR81896 and NMR152 were clustered together.

### 2.7. Performance of Selected Lines in Non-Stress (NS), Reproductive Stage Drought Stress (RS) and Vegetative Stage Submergence Stress (VS) Trials

Based on the multivariate analysis, ten BC_1_F_4_ lines were selected (Table 4). The selected lines had 72 to 82 DTF in the NS trial and 85 to 99 DTF in RS trial. The longest DTF in the RS trial was recorded for GEN239 but was earlier than MR219 (108 days). GEN140 recorded nine days earlier DTF than UKM5 in the NS trial but took two days longer than UKM5 in the RS trial of 2019MS. All lines had semi-dwarf PH in NS trials of 2018MS and 2019MS, which were almost similar to UKM5. In the RS trial, the PH of the lines was shorter but not drastically reduced. NP of GEN112, GEN133, GEN140 and GEN147 were also higher than UKM5 in 2019MS NS trial. However, NP of UKM5 was higher than most of the selected lines.

In the previous NS trial of 2018MS, the selected lines had a grain yield advantage of 98 to 3113 kg ha^−1^ over UKM5 and 290 to 4327 kg ha^−1^ over IR64-*Sub1*. Some of the selected lines had lower yield in the NS trial during the 2019MS compared to 2018MS and were lower than UKM5. GEN206 showed the highest yield advantage, followed by GEN230 and GEN239 over UKM5 in the NS trial of 2019MS. GEN239 and GEN206 also recorded high GY in the RS trial with a yield advantage of 2190.44 kg ha^−1^ and 1224.49 kg ha^−1^ over UKM5. Despite the lower yield recorded for GEN155 than UKM5 in the NS trial of 2019MS, this line had recorded yield advantage of 767.72 kg ha^−1^ over UKM5 in the RS trial. All selected lines showed higher GY than IR64-*Sub1* in the RS trial. In vs. trial, these lines showed higher SR than UKM5 and IR64-*Sub1* except GEN263 that was slightly lower than IR64-*Sub1*.

Evaluation under submergence stress showed great tolerance conferred by the selected lines (Table 5). The elongation percentage (EP) of the selected lines ranged from 6.79 to 55.80%, while CCC ranged from 7.33 to 20.37%. The selected lines except GEN263 had better survival under 14 days of submergence stress, which was 100% compared to lower survival recorded for IR64-*Sub1* (86.67%), UKM5 (66.67%) and the susceptible check, MR219 (40.00%).

## 3. Discussion

Despite being the most popular cultivated variety in Malaysia in the last 10–15 years, MR219 is lacking the tolerance to both drought and submergence stresses which hampers rice production. The present study reports the development of drought and submergence tolerant rice lines by combining the drought yield QTL (*qDTY*) and *Sub1* in the background of UKM5 and IR64-*Sub1*. UKM5 is an improved version of MR219 with two *qDTYs*; *qDTY_3_._1_* and *qDTY_12_._1_*. It is important to evaluate the morphological and agronomical traits related to yield; therefore, we also report the effect of a different combination of *qDTY* and *Sub1* on the phenotypic performance of the BC_1_F_4_ lines under normal and drought stress conditions. Selected lines from the population also were tested under 14 days submergence stress to evaluate the tolerance level. In the development of the experimental population, one backcrossing with UKM5 was carried out after selecting vigorous F_1_ individuals to develop the BC_1_F_1_ generation. Only one backcrossing was carried out as both parents were improved varieties and most likely free from any linkage drag. Past studies reported the effect of reduced yield under normal conditions caused by *qDTY_3_._1_* [19]. To ensure the advanced lines in each generation are free from any undesirable traits which may affect the yield or other traits, genotypic and phenotypic selection were carried out. This is important to ensure not only the advanced lines are carrying the desired QTLs but also by having favourable traits which are almost similar or better than the parents.

High variation among BC_1_F_4_ lines and checks were found for all traits in both NS and RS except NP in NS (Table S1). This high variation may have been caused by the effect of the presence of different QTL and QTL interaction in the genetic background [20]. The high differences among genotypes allowed an easier selection of genotypes with all the desired traits. The effect of RS to genotypes can be seen from the delayed DTF, reduced PH and other traits including GY compared to NS. The moderate to high *H* obtained for DTF, CC, PL, SPP, TGW and GY indicated less environmental variance and high variation among the tested genotypes that caused high genotypic variance. Blum [24] stated that the *H* of GY under stress conditions would be lower than that of GY under non-stress conditions; this will make the selection of GY less effective. However, the current study reported that a high *H* for GY indicates the high influence of genetic factors rather than the environment. Studies conducted in International Rice Research Institute (IRRI) also found moderate to high *H* for GY and found that the *H* of secondary traits was comparable under RS [14,18,25]. The high *H* of the yield-related traits serves as good predictors for GY and increase the reliability of selection based on these traits.

The effects of the QTL classes to any of the evaluated traits were different between NS and RS trials. Pleiotropic effects may also have been involved owing to the several traits influenced by a single *qDTY*, such as *qDTY_3_._1_* affecting FS, PH, SFP and TGW. Genes underlying this *qDTY* may be responsible for the expression of these traits under stress condition. Traits associated with the tolerance of drought stress, such as DTF, were improved by the presence of *qDTY_3_._1_*. Maisura et al. [26] and Ikmal et al. [27] reported the large effect of *qDTY_3_._1_* on DTF under moderate stress. Delay in flowering is one of the responses that plants showed to increasing water stress [19] but the presence of *qDTY* could have increased the ability of BC_1_F_4_ lines to tolerate drought stress and stabilised the DTF compared to MR219 which was delayed for 32 days. The non-significant differences found between the QTL classes for NP in the NS trial of both populations suggested that each combination of QTL had an almost similar effect on the production of panicles. Although significant differences were found in the RS trial, variations among the QTL combination were not that large to conclude the QTL combination that gave the best effect to NP. The significant positive correlation found between NP and GY in both NS and RS shows the importance of NP in determining GY.

In the RS trial, class F (*qDTY_3_._1_*) had the lowest PL compared with other classes and checks, but the positive complementary effect of *qDTY_3_._1_* and *Sub1* was observed on PL and SPP. Nevertheless, the effect of *Sub1* + *qDTY_3_._1_* to agronomical traits and GY was not as good as the effect of the single *qDTY_3_._1_*. Even though *qDTY_3_._1_* had shorter PL than *Sub1* + *qDTY_3_._1_*, the disadvantage was compensated by higher NP and FS. PL, SPP and FS are important in determining GY as they are closely related. Genotypes with high PL, SPP and FS would probably produce higher GY than genotypes with lower PL, SPP and FS. However, these traits must first be accompanied by higher NP for the genotype to produce high GY.

In this study, the combination of *Sub1* + *qDTY_3_._1_* + *qDTY_12_._1_* had better ability to compensate for the effect of stress on CC. Previously, Ikmal et al. [27] reported that the presence of *qDTY_12_._1_* and *qDTY_3_._1_* was able to maintain leaf rolling owing to the maintenance of plant water status while in other research, *qDTY_12_._1_* was found related to better root branching, transpiration and water uptake [28,29]. CC is affected by the availability of moisture and nutrients [30], therefore, the presence of *qDTY_12_._1_* and *qDTY_3_._1_* helps maintain the water status of plants, thus reducing chlorophyll degradation. Meanwhile, the highest reduction of PH was observed mostly in a class with a combination of *Sub1* + *qDTY_3_._1_* + *qDTY_12_._1_*. The reduction of PH is most likely due to the plant’s inability to maintain high turgor pressure and cell water potential [31]. By contrast, Fukao et al. [32] found that the expression of *Sub1A* under dehydration is high, which in turn increases the responsiveness of ABA and further limits water loss. Therefore, it is suggested that the presence of *Sub1* inhibits cell elongation by reducing the accumulation of ethylene under drought stress even though it remains unclear if the relationship between the production of ethylene and drought is species-specific [33].

Furthermore, the maintenance of high plant water status via the rooting abilities contributed by *qDTY_12_._1_* [28] was used for better transpiration and photosynthesis rate, which then contributed to a higher level of assimilates for grain filling, as observed by the high TGW for class A and other classes with *qDTY_12_._1_* in the NS trial. However, the effect of *qDTY_12_._1_* combination in the RS trial was significant only to NP and CC but not to other yield-related traits compared with the other combination. The expression of *qDTY_12_._1_* to the improvement of yield-related traits in RS trial might be lower because it is known that the expression of QTL is genetic-background dependent [34]. The differential effects of *qDTY*_2_._2_ originated from two different donors, namely, Kali Aus and Aday Sel were also reported [27]. Similar to previous studies, *qDTY_3_._1_* has a negative effect on GY under non-stress condition, which shown by the negative difference between classes with *qDTY_3_._1_* and the trial mean [19,20]. However, we are able to find a line with *Sub1* + *qDTY_3_._1_* that has almost similar GY to UKM5 and was better than IR64-*Sub1* in the NS trial. The combination of *Sub1* + *qDTY_12_._1_* was proved the most effective on GY in the NS trial because it showed the highest GY compared with the other QTL combinations. In the RS trial, the combination of *Sub1* + *qDTY_3_._1_* had the highest GY, followed by the combination of *Sub1* + *qDTY_12_._1_*. Therefore, the combination of either one of the *qDTY* with *Sub1* will lead to a better GY than if all QTLs were combined or were present as a single QTL in the RS trial. The expression of *Sub1*A is higher in tolerant genotypes under drought stress [32] which may explain the better performance of lines with *Sub1* under drought stress in this study.

From the trials, we have selected 10 lines with good phenotypes and GY by implementing the PCA and cluster analysis. Grouping of genotypes into different clusters enable the determination of genotypes with better performance by referring to the cluster means. The selected lines not only showed great tolerance to drought but also to submergence compared to UKM5 and other checks. The high tolerance level to submergence observed from the SR was contributed by the lower EP and CCC. Less elongation means the plant used less energy in the form of carbohydrate reserves for growth which was supposed to be used for resuming growth after submergence. The lower CCC recorded also plays an important role for the plant to maintain the optimal photosynthetic rate during and after submergence to produce enough photosynthetic products for energy and to recover.

## 4. Materials and Methods

### 4.1. Plant Materials

**Figure 4:**
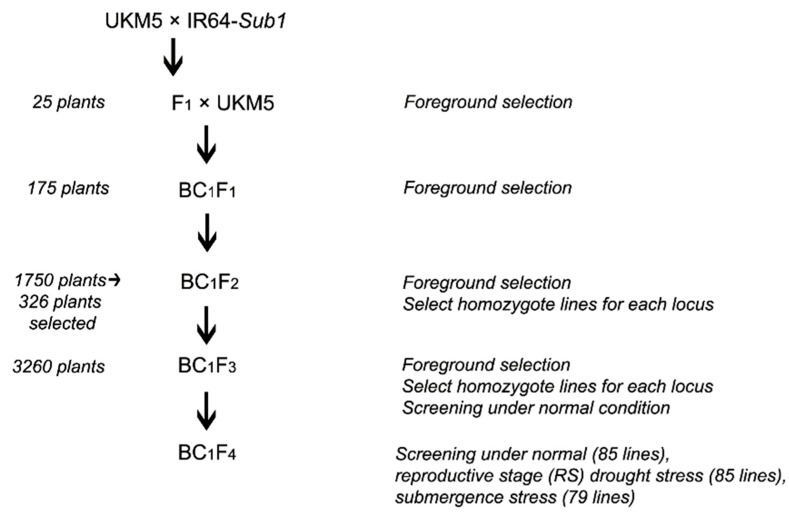
Crossing scheme for the development of BC_1_F_4_ lines.

A drought-tolerant line developed from the previous study conducted by Shamsudin et al. [12] was chosen as the recurrent parent. The seeds were deposited in Universiti Kebangsaan Malaysia’s seed storage room curated by Dr. Noraziyah Abd Aziz Shamsudin. The line which is designated as IR9784-226-335-1-5-1-1 (UKM5) possessing *qDTY_3_._1_* and *qDTY_12_._1_*, and recorded an average yield of 4327.07 to 10227.20 kg ha^−1^ under different water intensities in several experimental sites in Malaysia [35]. This line has a medium maturity period (90–110 days), semi-dwarf height (90–100 cm) and good grain cooking and eating qualities [36]. Another parent IR64-*Sub1*, the donor of *Sub1* were generated by introgressing submergence tolerance QTL (*Sub1*) from FR13A-derived line, IR40931-33-1-3-2 through a marker-assisted backcrossing strategy [37]. The seeds of IR64-*Sub1* were obtained from the International Rice Research Institute’s seed bank (Los Banos, Laguna, Philippines). The resulting IR64-*Sub1* was used as the donor for *Sub1* in this study. Instead of using the traditional donor of *Sub1* (FR13A) that is tall, photoperiod sensitive and has poor agronomical traits [38]. IR64-*Sub1* was chosen as it is most likely free from any undesirable traits. IR64-*Sub1* was crossed with UKM5 to develop F_1_ individuals followed by one back-crossing and selfing to develop the next generation (Figure 4). Selected BC_1_F_3_ lines were evaluated in the field under normal condition to select superior genotypes to be advanced to the BC_1_F_4_ generation. The selected BC_1_F_4_ lines were evaluated under normal, reproductive stage (RS) drought stress and vegetative stage submergence (VS) stress. Some of the selected BC_1_F_4_ lines may be evaluated under both RS and vs. or only in either one of the stresses.

### 4.2. Marker-Assisted Breeding and Genotyping

Plant DNA extraction was started by collecting the fresh leaf samples from the field and they were preserved in liquid nitrogen. DNA extraction procedure followed the CTAB protocol by Murray and Thompson [39] with some modifications. Leaf samples were ground inside 2.0 mL microcentrifuge tubes filled with 5 mm Qiagen stainless steel beads. The tubes were mounted on two Qiagen 24-well TissueLyser adapters and samples were ground for 1 min with 10 s break for every 30 s. In each tube, 500 µL CTAB buffer was pipetted and tubes were incubated in a 65 °C water bath for 30 min. Next, 500 μL of chloroform isoamyl alcohol mixture (24:1) was pipetted into the tubes and shaken for 5 min. The tubes were centrifuged at 16,100× *g* for 15 min to separate the mixture into layers. Then, 300 μL of the supernatant was pipetted into 1.5 μL microcentrifuge tubes containing 200 μL of isopropanol. The tubes were incubated in the freezer at −20 °C for 1 h to precipitate the DNA. After incubation, the tubes were centrifuged at 16,100× *g* for 15 min to allow DNA pellet formation at the base of the tubes. Isopropanol was drawn out from the tubes, and the DNA pellet was washed with 70% ethanol followed by drying at room temperature. The DNA pellet was suspended in 100 μL Tris-EDTA (TE; EDTA = Ethylenediaminetetraacetic acid) buffer added with 1 μL of RNAse prior to incubation in a water bath at 37 °C. The quality and quantity of DNA extracted were estimated using agarose gel and a Nanodrop Spectrophotometer. Polymorphic simple sequence repeat (SSR) markers linked to *qDTY_3_._1_* and *qDTY_12_._1_* together with Indel and Mismatch markers for *Sub1* were used for foreground selection (Table S8). Polymerase chain reaction (PCR) was conducted using fluorescently labelled primers following the method by Ikmal et al. [27] with some modifications. PCR mixture comprises 1.0 µL 10× Mg^2+^ free buffer, 0.2 µL of 25 mM MgCl_2_, 0.8 µL of 10 mM dNTPs, 0.5 µL of each 10 mM forward and reverse primers labelled with fluorescent dyes (HEX and FAM), 5.9 µL double deionized water, 1 µL of 25 ng/µL DNA template and 0.1 µL *Taq* DNA polymerase (5 U/µL). Amplification of the targeted DNA fragment was carried out using the Eppendorf Mastercylcer Nexus Gradient machine (Eppendorf, Hamburg, Germany). The polymerase chain reaction was started with 4 min of initial denaturation at 95 °C, followed by 35 cycles of denaturation for 45 s at 95 °C, 45 s of primer annealing at 55 to 60 °C depending on each primer used, 45 s of extension at 72 °C and final extension of 5 min at 72 °C. DNA fragment analysis using the Applied Biosystems Genetic Analyzer was carried out by Apical Scientific Sdn Bhd and the results of the PCR product sizes were interpreted using the GeneMapper^®^ analysis software.

### 4.3. Drought and Normal Screening Procedures

BC_1_F_3_ lines were evaluated only under non-stress condition during the 2018 main season (MS)—referred to as 2018MS. The selected 85 BC_1_F_4_ lines were arranged in a 7 × 13 Alpha Lattice design with two replications in both NS and RS trials during the 2019 main season (2019MS). For each BC_1_F_4_ line and checks (UKM5, IR64-*Sub1*, MR219, NMR152, IR81896, IR84984), 20 seedlings were transplanted in the field at a spacing of 25 cm between hills and 25 cm between rows. Drought stress was initiated at the 51st day after seeding (DAS) before the beginning of the reproductive stage and the field was maintained unflooded until maturity. Observational tube wells or known as piezometers were made from 1.2 m long × 2 inches diameter polyvinyl chloride (PVC) pipes to monitor soil water level throughout the drought stress period. The tubes were installed to a depth of 1 m with the extra 20 cm protruding above the soil surface. The water level was measured as the distance from the soil surface using a measuring stick. Irrigation by flash flooding for 24 h was given when the check varieties and 70% of the lines showed severe leaf wilting and the water level dropped to 100 cm [21]. The non-stress (NS) experiment was carried out in the irrigated and flooded field throughout the growing period.

### 4.4. Description of Field Management

Chemical fertilizer N:P:K, murate of potash (60% K_2_O), triple super phosphate (46% P_2_O_5_) and urea fertilizer (46% N) were applied according to a suggestion by the Department of Agriculture Malaysia. Carbacide (active ingredient 85% *w/w* carbaryl) was used to control insects while Almix 20WP (active ingredient 10% *w/w* metsulfuron-methyl and chlorimuron-ethyl 10% *w/w*) was used to control weeds. Mechanical control by hand-weeding was also undertaken a week after transplanting and during each growth stage. Benocide (active ingredient benomyl 50% *w/w*) was used for controlling blast, sheath blight and panicle rot diseases while Phyton 27 (active ingredient copper sulphate pentahydrate 27% *w/w*) was used to control brown spots, bacterial leaf blight and brown sheath diseases. All herbicides, insecticides and fungicides were applied according to the recommended amount as stated on their respective packaging.

### 4.5. Phenotypic Data Collection in RS and NS Experiments

In non-stress (NS) experiment of BC_1_F_3_ lines, only plant height (PH), days to 50% flowering (DTF), number of panicles per plant (NP) and grain yield (GY) were collected. In NS and reproductive stage drought stress (RS) experiments of BC_1_F_4_ lines, data collected were PH, DTF, NP, chlorophyll content (CC), panicle length (PL), number of spikelet per panicle (SPP), number of filled spikelet per panicle (FS), spikelet fertility percentage (SFP), thousand-grain weight (TGW) and GY. PH was measured from the root base to the highest panicle from three plants per plot. DTF was recorded by counting the number of days from seeding to the day for 50% of the plants in each plot had the panicle exerted. NP was counted from three plants in each plot and averaged. CC was measured 14 days after RS imposition (65 DAS) using the SPAD-502 chlorophyll meter (Konica Minolta) from the top, middle and bottom part of three leaves taken from five plants in each replication. Panicle length (PL) was measured from the panicle node to the tip of the uppermost spikelet of the panicle in cm. Three panicles from five plants were sampled randomly in each replication. SPP and FS were counted from the same panicle used to measure the PL and the proportion of FS from the overall SPP was converted to a percentage to obtain SFP. To obtain GY data, all plants from the plots were harvested and the data were normalized to 14% moisture content and converted to kg ha^−1^.

### 4.6. Submergence Screening Procedures

The screening for submergence tolerance was carried out following modified screening protocols from Das et al. [40] and Neeraja et al. [6]. Seventy-nine selected lines were seeded in pots and the number of seedlings was reduced to five by selecting seedlings with even height for each genotype. MR219 and UKM5 were used as the susceptible checks, while IR64-*Sub1* was used as the tolerant check. Randomized complete block design (RCBD) with three replications was used in this experiment. Twenty-one days after seeding, the plants were submerged in a PVC water tank measuring 1 × 1 × 1.5 m for 14 days and subsequently drained. The survival rate (SR) was measured seven days after water was drained using the formula:

SR = number of seedlings surviving after submergence/number of seedlings before submergence × 100(1)

### 4.7. Analysis of Variance (ANOVA), Broad-Sense Heritability (H) and Correlation

RStudio version 1.2.5001 was used to conduct ANOVA for the Alpha lattice design of RS and NS trials using the *PBIB.test* function in *Agricolae* package [41]. Statistical Tools for Agricultural Research (STAR) version 2.0.1 [42] provided by International Rice Research Institute was used to conduct ANOVA for the submergence experiment while Plant Breeding Tools (PBTools) version 1.4 [43] was used for computation of broad-sense heritability (*H)* of traits in NS and RS experiments. Pearson’s correlation coefficient values among traits were computed using RStudio version 1.2.5003 [44]. The built-in R package *Stats* [45] was used to obtain the correlation values while R package *Corrplot* [46] was used to visualize the correlation values into a graphical correlation matrix.

### 4.8. QTL Class Analysis

The selected BC_1_F_4_ lines were grouped into classes of every possible QTL combination after genotyping. The possible QTL combinations which were designated with different classes are presented in Table 6. Minitab 19 was used for the analysis using a mixed-model method following method [20,21,27,35]. In the mixed-model analysis method, restricted maximum likelihood (REML) was used for computation of variance while the degree of freedom was determined using the Satterthwaite method. The following model was used for the analysis:

*y*_ijkl_ = *µ* + *r*_k_ + *b(r)*_kl_ + *q*_i_ + *g(q)*_ij_ + *e*_ijkl_ (2)

where *µ* is the mean of the population, *r*_k_ is the effect of k^th^ replicate, *b(r)*_kl_ is the effect of the l^th^ block within the k^th^ replicate, *q*_i_ is the effect of i^th^ QTL, *g(q)*_ij_ is the effect of the j^th^ genotype nested within the i^th^ QTL while *e*_ijkl_ and *e*_ijk_ are the error terms for each equation, respectively. Fixed effects are QTLs and genotypes within QTLs while random effects are replicate and blocks within the replicate.

### 4.9. Multivariate Analysis

Principal component analysis (PCA) and cluster analysis were performed in R Studio version 1.2.5003 [44]. The *stats* package [45] was used to compute the PCA and was visualized using the *ggbiplot* package [47]. For the cluster analysis, *cluster* and *factoextra* packages [48,49] were used.

## 5. Conclusions

For many years, studies have been using popular QTL such as *Sub1*, *Saltol* and *Dro 1* to combat the drawbacks of abiotic stresses. In this study, we successfully demonstrated the effectiveness of combining *qDTY_12_._1_*, *qDTY_3_._1_* and *Sub1* in the background of UKM5 and IR64-*Sub1* using marker-assisted selection. The effect of *Sub1*, *qDTYs* and combination of the QTLs was confirmed in this study. These QTLs acted either synergistically or singly to cause effects on the morpho-physiological traits under submergence and drought stresses. However, the combination of three QTLs (*Sub1* + *qDTY_3_._1_* + *qDTY_12_._1_*) did not have a significant impact compared to the combination of two QTLs. Therefore, introgression or pyramiding of many QTLs into specific varieties would not always give positive results. The interaction of *Sub1* and *qDTYs* was found to improve the survival of the BC_1_F_4_ lines. It is also notable that *Sub1* also played an important role in tolerance to drought stress. Combination of *Sub1* + *qDTY_3_._1_* was the best combination for the improvement of grain yield under RS. The selected lines from this study can be developed to produce certified seeds for farmers in countries suffering from the reproductive stage drought stress and vegetative stage submergence stress to ensure the sustainability of rice production.

## Figures and Tables

**Table 1:** Range, mean, coefficient of variation (CV) and broad-sense heritability (*H*) recorded from non-stress (NS) and reproductive stage drought stress (RS) trials of BC_1_F_4_ lines. DTF, Days to flowering; PH, plant height; NP, number of panicles; CC, chlorophyll content; PL, panicle length; SPP, number of spikelets per panicle; FS, number of filled spikelets per panicle; SFP, spikelet fertility percentage; TGW, thousand-grain weight; GY, grain yield.

Traits	Range		Mean ± SE		CV (%)		Trial *H*	
	NS	RS	NS	RS	NS	RS	NS	RS
DTF	68.00–81.00	85.00–101.50	74.67 ± 0.34	91.09 ± 0.56	4.15	5.65	0.97	0.98
PH	68.00–114.50	73.00–103.00	104.07 ± 0.74	86.68 ± 0.79	6.52	8.38	0.75	1.00
NP	10.00–25.00	4.50–22.00	17.82 ± 0.39	10.26 ± 0.30	20.37	27.24	0.30	0.62
CC	31.50–50.00	18.90–47.10	42.08 ± 0.37	30.13 ± 0.60	8.14	18.28	1.00	0.98
PL	20.85–29.50	18.70–31.15	24.91 ± 0.18	23.96 ± 0.25	6.66	9.53	0.73	0.82
SPP	73.00–190.50	59.50–196.50	120.48 ± 2.38	119.51 ± 2.96	18.20	22.85	0.67	0.71
FS	47.50–158.50	36.00–160.00	94.80 ± 2.20	90.78 ± 2.79	21.36	28.39	0.61	0.73
SFP	48.61–98.07	39.06–91.96	78.99 ± 1.29	75.64 ± 1.28	15.00	15.63	0.75	0.56
TGW	22.80–27.70	17.40–26.85	25.98 ± 0.13	22.51 ± 0.24	4.51	9.83	0.75	0.86
GY	3617.00–13,893.00	378.50–4796.40	8046.00 ± 252.00	1457.50 ± 98.30	28.83	62.21	0.93	0.97

**Table 2:** Mean of quantitative trait loci (QTL) classes and checks for traits days to 50% flowering (DTF), plant height (PH), number of panicle per plant (NP), chlorophyll content (CC) and panicle length (PL) evaluated in non-stress (NS) and reproductive stage drought stress (RS) trials.

QTL Classes	QTL Combination	No. of Genotype	DTF (Days)		PH (cm)		NP		CC (SPAD)		PL (cm)	
			NS	RS	NS	RS	NS	RS	NS	RS	NS	RS
A	*Sub1* + *qDTY_3_._1_* +*qDTY_12_._1_*	2	75.00 cd	96.00 b	111.75 ab	83.50 ef	15.75 a	11.00 ab	42.10 bc	36.15 a	23.95 abc	24.68 ab
B	*Sub1* + *qDTY_3_._1_*	3	75.33 c	91.83 cd	106.17 bc	88.33 c	14.50 a	9.00 bcd	42.10 b	29.33 c	24.37 abc	26.50 a
C	*Sub1* + *qDTY_12_._1_*	12	75.13 c	90.08 d	104.96 c	87.54 c	18.58 a	9.38 bc	41.65 c	32.52 b	25.68 a	23.37 bc
E	*Sub1*	59	74.38 d	91.28 c	103.46 bc	86.31 d	18.11 a	10.47 ab	42.22 b	29.49 c	25.02 ac	24.13 b
F	*qDTY_3_._1_*	6	76.33 b	87.00 e	105.00 bc	88.17 c	18.25 a	10.67 ab	40.57 d	31.92 b	23.41 b	21.91 c
G	*qDTY_12_._1_*	3	76.50 b	95.67 b	105.83 abc	88.00 c	12.67 a	9.50 abcd	40.88 d	26.53 d	23.37 bc	24.12 abc
IR64-*Sub1*	*Sub1*	1	77.00 b	85.50 ef	107.00 bc	82.00 f	15.50 a	12.00 ab	37.55 e	20.30 e	23.80 abc	24.00 abc
MR219	-	1	75.50 bcd	107.50 a	101.00 abc	88.00 c	17.50 a	6.00 cd	34.90 f	31.20 bc	23.30 abc	25.75 ab
NMR152	-	1	83.00 a	84.00 f	112.00 abc	94.00 b	21.50 a	13.50 a	37.40 e	18.30 e	22.75 abc	24.90 abc
IR84984	*qDTY_12_._1_*	1	77.00 b	84.00 f	108.00 abc	85.00 de	17.00 a	5.50 d	40.35 d	25.60 d	26.35 abc	24.10 abc
IR81896	*qDTY_3_._1_*	1	75.50 bcd	85.50 ef	120.50 a	101.00 a	15.50 a	9.50 abcd	45.10 a	33.90 ab	25.00 abc	25.70 ab
UKM5	*qDTY_12_._1_*+ *qDTY_3_._1_*	1	77.00 b	84.00 f	107.50 abc	84.00 e	21.50 a	13.50 a	40.60 d	26.10 d	24.90 abc	24.45 abc
F-value			73.23	103.75	4.32	262.29	1.70	2.61	367.18	72.78	5.19	5.56
*p*-value			<0.001	<0.001	<0.001	<0.001	ns	<0.01	<0.001	<0.001	<0.001	<0.001
Trial mean			74.86	90.78	104.42	87.58	17.84	10.42	41.40	31.61	24.87	24.10

Mean values with the same letter at each column are not significantly different by Tukey’s honest significant difference (HSD) (*p* < 0.05).

**Table 3:** Mean of quantitative trait loci (QTL) classes and checks for traits number of spikelet per panicle (SPP), number of filled spikelet per panicle (FS), spikelet fertility percentage (SFP), thousand-grain weight (TGW) and grain yield (GY) evaluated in non-stress (NS) and reproductive stage drought stress (RS) trials.

QTL Classes	QTL Combination	No. of Genotype	SPP		FS		SFP (%)		TGW (g)		GY (kg ha^−1^)	
			NS	RS	NS	RS	NS	RS	NS	RS	NS	RS
A	*Sub1* + *qDTY_3_._1_* +*qDTY_12_._1_*	2	116.25 ab	111.25 bcd	75.50 ab	88.25 bcd	65.48 bc	78.70 a	26.95 a	21.45 cd	7418.64 ab	769.22 de
B	*Sub1* + *qDTY_3_._1_*	3	116.67 ab	151.67 ab	74.33 b	117.17 ab	64.44 c	76.35 a	25.40 b	24.02 abc	6551.29 bc	2427.45 a
C	*Sub1* + *qDTY_12_._1_*	12	130.96 a	106.13 d	101.79 ab	78.63 d	78.90 ab	75.77 a	25.63 b	22.50 bcd	8678.17 a	1839.25 b
E	*Sub1*	59	118.92 ab	122.91 c	94.07 ab	93.33 bc	79.32 ab	75.26 a	25.98 b	22.41 bcd	8136.75 a	1380.91 c
F	*qDTY_3_._1_*	6	124.33 ab	104.92 cd	106.17 a	83.17 cd	84.71 a	79.45 a	26.53 ac	23.61 abc	7680.65 ab	1448.10 c
G	*qDTY_12_._1_*	3	103.33 b	108.83 cd	82.33 ab	79.67 cd	79.73 abc	72.19 a	26.13 abc	21.45 d	4514.37 d	943.09 de
IR64-*Sub1*	*Sub1*	1	93.00 ab	86.50 cd	85.50 ab	71.00 bcd	92.01 a	82.17 a	25.80 abc	21.60 bcd	6938.88 abc	342.66 e
MR219	-	1	89.00 ab	134.00 abcd	81.50 ab	118.50 abcd	91.55 a	89.71 a	26.25 abc	25.95 a	5959.82 bcd	951.32 cde
NMR152	-	1	93.00 ab	116.00 bcd	76.50 ab	90.00 abcd	82.12 abc	78.62 a	25.35 bc	26.15 a	9756.00 a	2290.12 ab
IR84984	*qDTY_12_._1_*	1	124.00 ab	119.50 bcd	98.00 ab	95.00 abcd	80.18 abc	78.82 a	26.60 abc	25.25 ab	4519.04 cd	1122.43 cd
IR81896	*qDTY_3_._1_*	1	100.00 ab	191.50 a	90.00 ab	148.00 a	89.96 a	77.25 a	26.65 abc	20.50 d	4346.40 cd	2690.46 a
UKM5	*qDTY_12_._1_*+ *qDTY_3_._1_*	1	147.00 ab	139.50 abcd	111.50 ab	103.00 abcd	75.89 abc	74.26 a	26.20 abc	26.05 a	9625.60 a	2605.91 a
F-value			3.42	6.47	2.68	4.78	4.32	0.61	2.23	8.84	21.30	45.73
*p*-value			<0.01	<0.001	<0.01	<0.001	<0.001	ns	<0.05	<0.001	<0.001	<0.001
Trial mean			119.63	116.64	94.52	87.20	79.40	73.99	25.99	23.12	7967.26	1560.33

Mean values with the same letter at each column are not significantly different by Tukey’s HSD (*p* < 0.05).

**Table 4:** Selected lines and the mean of days to 50% flowering (DTF), plant height (PH), number of panicle per plant (NP) and grain yield (GY) in non-stress (NS) and reproductive stage drought stress (RS) trials in 2018MS and 2019MS.

Genotype	QTL	DTF (Days)			PH (cm)			NP			GY (kg ha^−1^)		
		2018MS	2019MS		2018MS	2019MS		2018MS	2019MS		2018MS	2019MS	
		NS	NS	RS	NS	NS	RS	NS	NS	RS	NS	NS	RS
GEN112	*Sub1* + *qDTY_3_._1_*	79	80	89	105	103.00	97	23	18	9	11,485	8488.00	2590.58
GEN133	*qDTY_3_._1_*	82	81	85	107	104.00	83	26	22	12	9518	8645.00	2557.95
GEN140	*Sub1*	70	68	87	101	97.00	84	24	21	13	10,875	7582.00	2949.48
GEN147	*Sub1*	70	71	89	105	103.00	83	25	19	11	10,753	7565.00	2753.72
GEN155	*Sub1*	76	75	87	99	103.50	93	17	16	12	7448	7062.00	3373.63
GEN206	*Sub1* + *qDTY_12_._1_*	75	77	92	102	108.00	84	18	23	14	8470	13,893.00	3830.40
GEN219	*Sub1* + *qDTY_12_._1_*	75	77	90	104	107.50	73	14	15	8	6700	8996.30	2607.22
GEN230	*Sub1* + *qDTY_12_._1_*	73	75	86	110	109.00	95	17	25	12	6538	13,015.00	2475.13
GEN239	*Sub1*	72	76	99	104	105.00	86	21	23	14	10,480	11,084.00	4796.35
GEN263	*Sub1* + *qDTY_12_._1_*	75	72	87	104	100.50	88	21	19	11	8485	8541.00	2758.81
IR64-*Sub1*	*Sub1*	80	77	86	103	107.00	82	18	16	12	7158	6938.88	342.66
IR81896	*qDTY_3_._1_*	-	76	86	-	120.50	101	-	16	10	-	4346.40	2690.46
IR84984	*qDTY_12_._1_*	-	77	84	-	108.00	85	-	17	6	-	4519.04	1122.43
MR219	*-*	74	76	108	98	101.00	88	16	17	6	6374	5959.82	951.32
NMR152	*-*	-	83	84	-	112.00	94	-	22	14	-	9756.00	2290.12
UKM5	*qDTY_3_._1_* + *qDTY_12_._1_*	78	77	84	110	107.50	84	21	22	14	8372	9625.60	2605.91

**Table 5:** Selected lines and the mean of elongation percentage (EP), percentage of change of chlorophyll content (CCC) and survival rate (SR) in vegetative stage submergence stress (VS) trial.

Genotype	QTL	EP (%)	CCC (%)	SR (%)
GEN112	*Sub1* + *qDTY_3_._1_*	6.79	9.42	100.00
GEN140	*Sub1*	55.80	16.01	100.00
GEN147	*Sub1*	49.44	12.06	100.00
GEN155	*Sub1*	25.07	20.37	100.00
GEN206	*Sub1* + *qDTY_12_._1_*	36.94	15.33	100.00
GEN219	*Sub1* + *qDTY_12_._1_*	19.62	15.84	100.00
GEN230	*Sub1* + *qDTY_12_._1_*	27.05	7.33	100.00
GEN239	*Sub1*	34.47	11.03	100.00
GEN263	*Sub1* + *qDTY_12_._1_*	31.94	15.16	80.00
UKM5	*qDTY_12_._1_* + *qDTY_3_._1_*	54.33	27.90	66.67

**Table 6:** Possible QTL classes for UKM5*/IR64-*Sub1* population in NS, RS and vs. trials.

QTL Class Designation	QTL Combination
A	*Sub1* + *qDTY_3_._1_* + *qDTY_12_._1_*
B	*Sub1* + *qDTY_3_._1_*
C	*Sub1* + *qDTY_12_._1_*
D	*qDTY_3_._1_* + *qDTY_12_._1_*
E	*Sub1*
F	*qDTY_3_._1_*
G	*qDTY_12_._1_*

## Data Availability

The data presented in this study are available on request from the corresponding author. The data are not publicly available due to future work is under planning.

## Supplementary Materials

The following are available online at https://www.mdpi.com/2223-7747/10/2/225/s1, Figure S1: Abiotic parameters measured during the imposition of reproductive stage drought stress, Table S1: Results of ANOVA on traits evaluated in NS trial for UKM5*/IR64-*Sub1* population, Table S2: Results of ANOVA on traits evaluated in RS trial for UKM5*/IR64-*Sub1* population, Table S3: Mean of traits (DTF, PH, NP, CC and PL) in NS and RS experiment, Table S4: Mean of traits (SPP, FS, SFP, TGW and GY) in NS and RS experiment, Table S5: Principal component analysis results for NS trial of UKM5*/IR64-*Sub1* population: Cluster means for traits evaluated in non-stress (NS) and reproductive stage drought stress (RS) trials in 2019MS, Table S7. Principal component analysis results for RS trial of UKM5*/IR64-*Sub1* population, Table S8: Details of marker used for genotyping
