# Supplementary material for: Incorporating Drought and Submergence Tolerance QTL in Rice (Oryza sativa L.)—The Effects under Reproductive Stage Drought and Vegetative Stage Submergence Stresses
Source: Plants (Basel). 2021 Jan 24;10(2):225. doi: 10.3390/plants10020225 (PMC7912027; doi:10.3390/plants10020225)
Supplement: Supplementary file 1 [file plants-10-00225-s001.pdf]

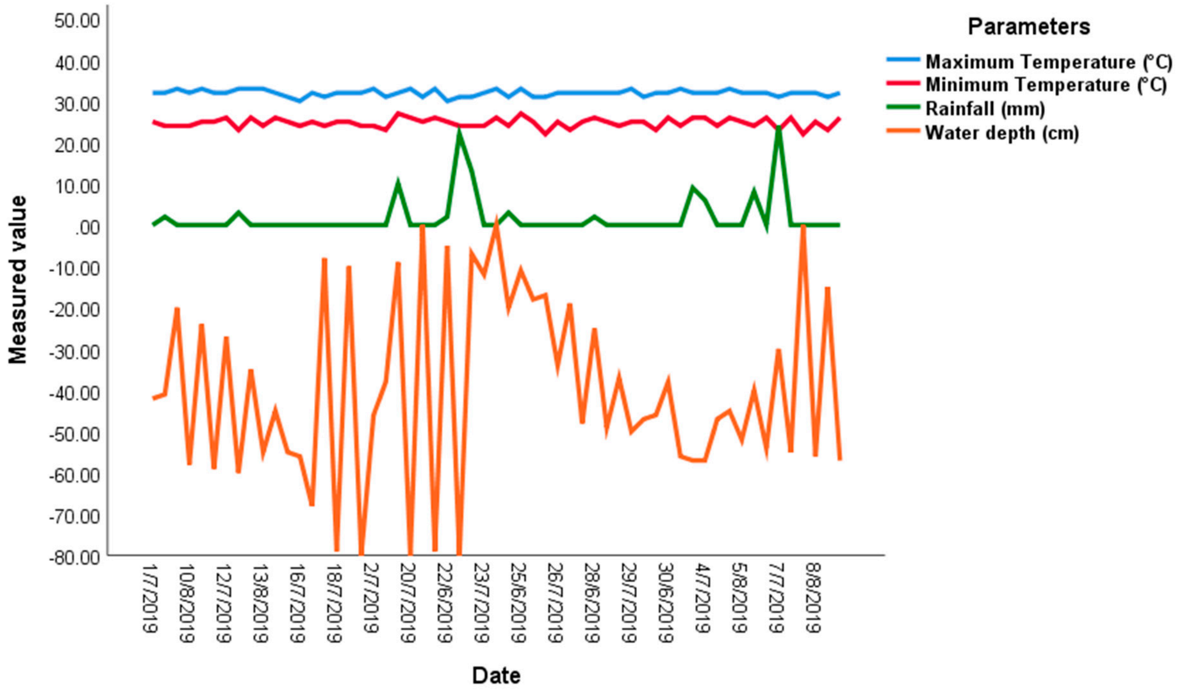

Figure S1. Abiotic parameters measured during the imposition of reproductive stage drought stress

Table S1. Results of ANOVA on traits evaluated in NS trial for UKM5*/IR64-*Sub1* population.

Source of variation	DF	DTF	PH	NP	CC	PL	SPP	FS	SFP	TGW	GY
Genotype	90	19.76 ***	93.98 ***	25.45 ns	24.22 ***	5.37 ***	977.14 ***	785.42 ***	272.25 ***	2.59 ***	10855980.00 ***
Block	24	0.43	22.93	15.31	0.07	1.17	238.83	325.28	113.34	0.62	812831.00
Rep	1	0.02	33.43	6.73	0.50 **	0.02	13.19	186.02	161.93	0.49	232147.00
Error	66	0.27	23.40	19.90	0.06	1.61	356.04	329.96	54.69	0.66	749474.00
CV (%)		0.70	4.60	24.10	0.60	4.90	15.00	18.90	10.60	3.00	10.90
Trial mean		74.86	104.42	17.84	41.40	24.87	119.63	94.52	79.40	25.99	7967.26

Days to flowering (DTF), plant height (PH), number of panicles (NP), chlorophyll content (CC), panicle length (PL), number of spikelet per panicle (SPP), number of filled spikelet per panicle (FS), spikelet fertility percentage (SFP), thousand-grain weight (TGW), grain yield (GY), degree of freedom (DF), Coefficient of variation (CV) in %, non-significant (ns).

, * indicate significant at $p < 0.01$ and $p < 0.001$, respectively

Table S2. Results of ANOVA on traits evaluated in RS trial for UKM5*/IR64-*Sub1* population.

Source of variation	DF	DTF	PH	NP	CC	PL	SPP	FS	SFP	TGW	GY
Genotype	90	60.06 ***	104.85 ***	16.03 ***	62.89 ***	9.88 ***	1544.62 ***	1340.08 ***	266.71 ***	14.30 ***	1648217.00 ***
Block	24	1.00	0.27	4.93	0.82	2.31	336.96	365.54	138.26	1.74	41421.00
Rep	1	5.28	41.78 ***	66.48 **	5.73 *	3.71	2051.21 *	1335.43	4.14	4.68	1968.00
Error	66	1.38	0.29	6.73	1.43	1.78	434.43	359.90	116.91	1.46	51825.00
CV (%)		1.20	0.60	23.70	3.40	5.80	16.80	20.70	14.50	5.20	14.60
Trial mean		90.78	87.58	10.42	31.61	24.10	116.64	87.20	73.99	23.12	1560.33

Days to flowering (DTF), plant height (PH), number of panicles (NP), chlorophyll content (CC), panicle length (PL), number of spikelet per panicle (SPP), number of filled spikelet per panicle (FS), spikelet fertility percentage (SFP), thousand-grain weight (TGW), grain yield (GY). DF = Degree of freedom, CV = Coefficient of variation (%)

*, **, *** indicate significant at $p < 0.05$, $p < 0.01$ and $p < 0.001$, respectively

Table S3. Mean of traits (DTF, PH, NP, CC and PL) in NS and RS experiment.

Genotype	DTF		PH		NP		CC		PL	
	NS	RS	NS	RS	NS	RS	NS	RS	NS	RS
GEN112	80.00	89.00	103.00	97.00	18.00	8.50	44.40	27.50	23.75	26.65
GEN113	80.00	85.00	105.00	94.00	21.00	6.00	41.00	37.20	25.75	23.10
GEN114	80.00	90.50	107.50	98.00	20.00	10.00	43.40	23.30	25.55	26.95
GEN116	80.00	85.00	106.00	96.00	15.50	11.50	41.30	26.60	24.45	22.70
GEN117	80.00	88.00	108.00	89.00	13.00	14.00	45.10	20.80	23.75	24.05
GEN118	75.00	88.00	102.50	85.00	18.50	11.00	41.30	30.50	24.95	25.65
GEN122	78.00	90.00	100.00	92.00	17.00	9.50	42.20	26.20	27.30	25.70
GEN124	77.50	90.50	95.00	103.00	25.00	11.00	47.70	37.00	24.55	22.05
GEN125	77.50	87.50	99.50	85.00	13.50	11.50	40.00	35.10	25.80	25.00
GEN127	78.00	88.00	113.50	89.00	19.00	7.50	45.50	24.50	25.25	23.85
GEN128	78.00	90.00	113.50	91.00	20.50	9.50	39.90	24.60	28.00	21.70
GEN129	78.00	85.00	103.50	87.00	13.50	10.00	46.00	24.85	25.95	23.45
GEN132	81.00	90.50	113.50	82.00	22.50	11.00	45.10	22.80	25.60	26.45
GEN133	81.00	85.00	104.00	83.00	22.00	11.50	37.40	30.30	21.60	21.50
GEN134	78.00	85.50	105.00	74.00	14.00	10.00	39.50	24.20	25.45	24.80
GEN138	71.00	85.50	103.50	82.00	10.50	4.50	38.60	29.00	25.80	27.20
GEN140	68.00	86.50	97.00	84.00	21.00	12.50	43.10	27.20	23.95	22.90
GEN146	68.00	100.50	99.50	79.00	14.50	6.00	45.80	27.00	24.50	25.45
GEN147	71.00	89.00	103.00	83.00	19.00	11.00	42.80	34.50	28.00	26.50
GEN148	71.00	100.00	105.50	85.00	17.00	9.50	42.00	28.50	25.20	31.15
GEN150	73.00	87.00	101.00	79.00	17.00	8.50	42.00	32.50	24.00	22.10
GEN152	69.00	101.00	97.50	79.00	15.50	11.50	40.90	30.30	23.80	20.10
GEN154	69.00	101.50	107.00	87.00	22.50	13.00	38.20	31.60	25.35	25.35
GEN155	74.50	86.50	103.50	93.00	15.50	11.50	42.50	35.20	25.70	25.20
GEN157	73.50	88.00	103.50	101.00	23.50	14.00	40.50	21.30	25.00	27.25
GEN158	74.00	98.50	110.50	98.00	18.00	11.50	42.30	26.30	26.50	26.75

GEN159	74.00	100.00	107.50	87.00	17.00	6.00	42.80	32.00	26.30	25.85
GEN160	68.00	89.50	110.00	89.00	11.00	12.00	40.00	30.30	26.65	22.75
GEN163	75.00	87.00	100.00	96.00	22.50	10.50	42.00	28.90	25.85	23.15
GEN164	74.00	86.50	101.00	78.00	19.00	13.50	42.00	28.80	25.80	24.40
GEN165	74.00	101.50	111.00	74.00	15.50	9.00	36.00	23.35	24.60	25.25
GEN166	74.50	98.00	106.50	75.00	15.50	12.50	46.30	33.90	22.95	24.60
GEN167	75.00	98.00	110.00	96.00	14.00	10.50	44.70	25.50	26.60	24.80
GEN168	74.00	101.00	111.50	85.00	19.00	10.00	38.80	32.00	25.75	22.10
GEN170	70.00	87.00	68.00	77.00	12.50	8.50	46.40	18.90	27.40	24.05
GEN172	70.00	89.00	99.50	75.00	19.00	7.50	43.60	22.70	24.35	21.65
GEN176	73.00	89.50	109.00	79.00	22.00	9.50	43.10	27.00	23.50	22.95
GEN185	72.00	89.50	102.00	84.00	15.00	9.50	45.30	29.70	24.15	20.75
GEN187	77.00	89.00	114.00	95.00	17.50	6.50	38.60	32.70	26.60	22.25
GEN188	77.00	89.50	102.00	100.00	16.00	9.50	46.10	34.70	29.50	22.95
GEN190	77.00	87.00	102.00	78.00	20.00	9.50	44.20	31.30	27.00	23.55
GEN191	75.00	87.00	93.00	79.00	23.00	13.00	44.10	30.50	27.55	21.95
GEN192	75.00	95.50	103.50	86.00	16.50	12.00	44.10	29.50	26.00	27.25
GEN193	75.00	94.00	111.50	83.00	16.00	10.50	46.10	33.20	23.75	24.80
GEN197	68.00	86.50	91.00	85.00	18.00	10.00	46.70	31.30	22.75	24.45
GEN204	76.00	101.00	110.00	88.00	14.50	5.50	39.85	39.20	23.10	22.50
GEN206	76.50	91.50	108.00	84.00	23.00	14.00	41.40	21.30	25.95	25.30
GEN207	68.00	91.50	91.50	88.00	19.00	10.00	50.00	20.80	23.45	19.35
GEN209	76.00	91.50	110.50	95.00	17.50	12.00	47.50	34.40	24.50	18.70
GEN210	75.00	89.00	106.50	95.00	18.50	11.00	38.20	34.00	20.85	19.05
GEN213	75.00	86.00	99.50	93.00	19.50	6.50	37.70	31.20	24.05	21.15
GEN215	77.00	98.50	110.00	91.00	10.00	5.00	41.65	26.40	23.70	24.00
GEN216	72.00	96.00	103.00	78.00	14.50	11.00	43.70	25.80	28.80	24.00
GEN218	74.50	89.50	104.00	76.00	18.00	13.50	49.10	34.50	24.50	21.70
GEN219	76.50	89.50	107.50	73.00	15.00	8.00	37.60	34.80	26.00	24.00
GEN220	76.50	87.00	98.00	74.00	11.00	13.50	36.00	28.60	21.55	21.90

GEN222	75.50	98.00	105.00	85.00	11.50	8.50	38.47	32.20	24.35	26.75
GEN223	75.00	101.00	112.00	86.00	15.00	14.00	43.30	31.50	23.55	25.65
GEN226	77.00	95.50	98.50	97.00	14.00	10.50	40.50	31.30	25.90	25.20
GEN227	75.00	98.00	112.00	84.00	15.50	11.50	38.10	39.10	24.15	24.55
GEN228	77.00	86.50	98.00	85.00	13.50	17.50	41.50	23.80	22.80	25.00
GEN229	75.00	87.00	89.50	86.00	20.50	6.50	46.60	30.70	24.75	23.25
GEN230	75.00	86.00	109.00	95.00	25.00	11.50	44.70	36.10	23.55	23.45
GEN231	74.50	86.50	100.00	85.00	20.50	6.00	42.80	31.20	25.10	25.05
GEN234	76.50	86.50	97.50	84.00	24.50	14.00	43.20	47.10	21.50	24.55
GEN236	74.50	86.50	101.00	75.00	14.50	11.00	46.30	27.30	24.80	24.75
GEN238	72.50	87.00	109.50	87.00	18.50	10.50	43.70	36.50	25.00	25.55
GEN239	75.50	99.50	105.00	86.00	23.00	13.50	43.50	28.70	24.00	23.90
GEN240	75.00	95.50	101.00	88.00	18.00	7.00	39.10	41.80	23.80	22.60
GEN242	76.00	101.50	109.50	99.00	17.00	10.00	45.00	24.60	24.85	26.45
GEN243	75.50	89.00	102.00	97.00	16.00	10.50	43.80	23.60	26.90	24.50
GEN247	76.50	93.00	106.00	87.00	19.50	6.50	45.50	30.70	23.50	22.55
GEN249	75.50	92.50	114.50	85.00	23.50	8.50	37.60	29.30	24.90	28.80
GEN250	75.50	86.50	99.00	89.00	19.00	10.00	31.50	31.20	20.90	20.90
GEN251	76.00	93.00	105.50	90.00	20.50	9.00	35.50	20.80	22.00	24.40
GEN258	76.50	93.00	108.50	89.00	18.00	8.50	38.60	20.60	25.10	21.00
GEN261	73.00	93.00	103.50	93.00	15.00	12.00	39.90	38.30	25.60	25.05
GEN262	72.00	89.00	104.00	94.00	19.00	8.50	43.90	41.30	26.30	26.50
GEN263	72.00	87.00	100.50	88.00	19.00	11.50	41.00	36.10	24.60	19.55
GEN264	74.00	86.50	108.50	85.00	16.50	22.00	39.60	32.10	24.90	27.75
GEN266	72.00	86.50	109.50	84.00	23.00	12.50	35.40	37.40	25.65	22.35
GEN267	71.00	93.00	110.00	73.50	18.50	11.00	40.20	29.60	24.00	22.35
GEN268	73.50	86.50	99.50	82.00	21.50	7.50	45.20	34.70	24.30	24.20
GEN269	73.50	93.00	107.00	90.50	25.00	9.00	40.30	32.35	24.90	20.05
GEN270	72.00	89.00	108.00	87.50	13.50	7.00	40.00	35.30	26.80	25.25
IR64-*Sub1*	77.00	85.50	107.00	82.00	15.50	12.00	37.55	20.30	23.80	24.00

IR81896	75.50	85.50	120.50	101.00	15.50	9.50	45.10	33.90	25.00	25.70
IR84984	77.00	84.00	108.00	85.00	17.00	5.50	40.35	25.60	26.35	24.10
MR219	75.50	107.50	101.00	88.00	17.50	6.00	34.90	31.20	23.30	25.75
NMR152	83.00	84.00	112.00	94.00	21.50	13.50	37.40	18.30	22.75	24.90
UKM5	77.00	84.00	107.50	84.00	21.50	13.50	40.60	26.10	24.90	24.45
$LSD_{0.05}$	8.87	15.47	19.36	20.44	10.07	7.99	9.83	15.83	4.62	6.28

Table S4. Mean of traits (SPP, FS, SFP, TGW and GY) in NS and RS experiment

Genotype	SPP		FS		SFP		TGW		GY	
	NS	RS	NS	RS	NS	RS	NS	RS	NS	RS
GEN112	103.50	157.50	70.50	124.50	67.93	79.07	23.50	24.10	8488.00	2590.58
GEN113	143.00	140.50	96.00	96.50	67.62	62.22	25.90	24.20	9748.00	475.79
GEN114	93.50	141.00	86.00	129.50	91.47	91.84	26.50	21.40	12163.50	828.72
GEN116	184.00	129.00	158.50	115.50	86.11	89.53	26.10	22.55	7006.00	939.12
GEN117	73.00	136.50	55.50	123.50	77.03	90.47	26.45	22.90	5005.00	1807.53
GEN118	127.00	125.50	118.00	92.50	92.86	74.18	24.85	18.40	8381.00	1189.52
GEN122	144.00	118.00	87.50	102.00	62.40	86.45	26.20	21.95	8362.00	2074.84
GEN124	98.00	121.50	82.00	68.00	82.74	55.90	27.70	17.40	11155.00	663.52
GEN125	135.50	133.50	114.50	102.50	83.73	76.81	26.40	21.45	5294.00	861.35
GEN127	121.50	111.00	92.00	61.50	75.94	56.13	26.20	24.15	5522.00	611.29
GEN128	144.50	127.00	95.00	86.50	65.85	68.59	27.00	23.70	11062.00	670.78
GEN129	115.50	160.50	91.50	85.00	78.57	54.00	26.05	19.65	4535.00	740.40
GEN132	123.00	135.00	96.00	88.00	80.14	65.13	26.80	26.00	8509.00	804.97
GEN133	117.50	88.00	109.50	79.00	93.18	89.76	26.70	24.05	8645.00	2557.95
GEN134	154.50	140.00	124.50	100.00	80.62	72.71	26.80	19.95	6682.00	684.44
GEN138	135.00	162.50	74.00	134.50	55.00	82.77	26.40	26.15	5219.00	3928.29
GEN140	105.00	83.50	98.00	69.00	93.36	82.36	26.40	24.65	7582.00	2949.48
GEN146	135.00	145.00	101.00	119.00	74.77	82.17	23.00	19.55	5687.00	378.47
GEN147	161.00	139.50	146.50	111.00	91.02	79.63	25.05	24.35	7565.00	2753.72
GEN148	123.00	196.50	90.50	160.00	73.75	81.35	26.60	24.00	5782.00	2202.87
GEN150	120.50	114.00	108.00	99.50	89.56	87.50	26.60	23.35	8657.00	1774.91
GEN152	145.50	79.00	127.00	66.00	87.22	83.75	25.95	22.40	7073.00	1154.99
GEN154	115.00	153.50	108.00	130.50	93.12	85.01	26.80	24.70	11101.00	1732.35
GEN155	122.00	118.00	79.50	90.00	65.00	76.14	27.20	25.80	7062.00	3373.63
GEN157	114.00	165.00	93.00	144.00	82.21	87.12	26.90	21.80	11958.00	1869.99
GEN158	116.50	129.00	88.50	104.00	75.90	81.37	26.30	22.45	7496.00	1187.62

GEN159	121.50	148.50	78.50	122.50	65.13	82.49	27.15	20.05	9617.00	703.57
GEN160	113.00	125.50	107.00	91.50	94.87	72.91	26.80	22.40	5817.00	1154.99
GEN163	127.00	126.50	109.50	109.00	86.20	86.40	22.80	22.15	8631.00	1862.96
GEN164	138.50	147.00	126.00	116.00	90.71	78.86	24.35	23.15	7927.00	1644.40
GEN165	129.50	147.50	100.50	131.00	77.61	88.76	26.05	22.50	7721.00	1220.25
GEN166	92.00	112.50	79.00	102.00	85.70	90.65	23.70	22.45	6136.00	1187.62
GEN167	135.00	107.00	111.50	90.00	82.94	83.92	24.60	22.90	7102.00	779.92
GEN168	128.00	87.00	94.00	65.50	72.91	75.98	25.55	18.75	8437.00	643.26
GEN170	114.50	106.50	113.00	81.00	98.06	75.83	23.30	18.60	6037.10	1324.65
GEN172	115.00	97.50	97.00	82.50	84.54	85.50	27.15	22.95	8078.00	1513.89
GEN176	108.50	124.00	82.50	91.00	76.55	73.39	23.45	22.75	8948.00	1383.38
GEN185	129.50	91.00	63.00	67.00	48.61	73.18	23.80	18.05	4458.00	645.14
GEN187	183.50	101.00	96.50	79.00	52.36	77.15	22.80	19.35	5451.00	673.07
GEN188	190.50	97.00	135.50	81.00	71.22	83.67	25.25	18.35	8579.00	826.33
GEN190	97.50	134.00	76.50	104.00	75.39	77.63	25.95	22.50	11443.00	1884.96
GEN191	152.50	99.00	134.50	73.50	88.07	74.17	26.50	21.45	12732.80	1486.54
GEN192	157.00	175.00	100.50	134.00	64.01	79.29	25.60	21.15	10607.00	1172.30
GEN193	120.00	99.50	61.00	73.50	50.80	73.73	27.40	22.25	7617.00	853.28
GEN197	106.00	119.50	97.50	102.00	92.16	85.36	22.80	24.20	7748.00	1246.44
GEN204	101.00	84.00	95.50	67.00	94.94	79.57	26.45	25.90	5545.00	530.91
GEN206	115.50	109.50	90.50	80.00	77.37	73.43	26.15	26.50	13893.00	3830.40
GEN207	107.00	86.50	102.00	69.00	95.35	80.02	25.80	21.30	8861.00	587.83
GEN209	101.50	63.50	74.50	46.50	73.63	72.10	26.25	21.60	6658.14	632.96
GEN210	95.50	71.50	77.50	36.00	81.70	50.53	26.50	23.60	9545.86	517.52
GEN213	121.50	79.00	63.50	62.00	51.00	76.67	26.65	24.10	10201.00	702.14
GEN215	99.00	86.00	68.50	56.00	72.00	64.56	26.30	23.05	3616.50	564.87
GEN216	165.50	143.00	122.50	91.50	73.83	64.03	27.20	25.75	7384.00	3341.00
GEN218	130.00	98.50	116.50	76.00	89.50	77.00	25.80	21.85	7709.00	1367.40
GEN219	109.50	106.00	89.50	96.00	81.81	90.80	26.50	23.75	8996.30	2607.22
GEN220	93.50	108.50	71.00	92.50	75.85	85.15	26.20	20.50	4866.00	1326.31

GEN222	126.50	160.50	84.00	128.00	66.77	79.76	27.05	21.55	5510.00	1013.99
GEN223	111.50	135.00	78.50	92.50	70.40	67.21	26.30	21.80	5947.00	763.47
GEN226	95.50	111.00	69.00	72.00	74.85	64.07	26.40	22.55	5232.00	1126.31
GEN227	112.50	123.00	90.00	103.00	80.16	83.68	26.50	20.65	7219.90	685.17
GEN228	116.50	138.50	103.00	111.00	88.59	80.00	25.00	20.25	7104.00	1691.89
GEN229	122.50	121.00	76.50	47.00	62.72	39.06	26.70	20.60	9761.10	459.16
GEN230	124.50	95.50	102.00	75.00	81.97	78.53	26.20	25.75	13015.00	2475.13
GEN231	150.50	158.00	118.50	89.50	77.96	60.50	25.40	20.45	7849.00	2744.29
GEN234	106.50	95.50	101.00	44.50	94.83	46.63	26.90	22.95	12910.00	1187.62
GEN236	92.00	139.50	76.50	107.00	82.97	76.72	25.40	24.40	6518.00	1944.27
GEN238	120.00	129.50	104.00	71.00	86.28	54.89	26.60	24.00	6676.00	833.67
GEN239	94.50	110.50	65.00	95.50	68.48	86.41	26.60	26.55	11084.00	4796.35
GEN240	106.00	95.00	98.50	53.50	92.94	58.23	26.60	23.55	7869.80	556.26
GEN242	117.50	132.00	107.50	90.50	91.34	66.86	25.90	20.80	5061.00	938.09
GEN243	102.00	132.50	88.50	97.50	84.10	71.59	26.30	20.30	7029.00	809.27
GEN247	101.50	86.50	74.00	57.00	72.97	65.64	26.20	23.85	8132.00	1448.64
GEN249	90.00	143.00	65.50	127.50	70.85	89.16	26.50	24.85	13191.00	1433.45
GEN250	102.50	105.50	82.50	87.50	79.47	82.91	26.90	26.10	8442.00	1950.01
GEN251	92.50	142.50	47.50	108.00	51.47	75.93	26.75	23.75	10113.00	939.37
GEN258	110.50	97.50	90.50	52.00	82.55	43.69	26.70	18.20	6153.00	449.27
GEN261	101.00	151.50	91.50	100.50	89.98	66.28	26.40	25.05	7957.00	1430.11
GEN262	126.00	138.00	96.50	123.00	76.61	89.43	27.20	23.15	8183.00	2867.86
GEN263	116.00	74.00	107.00	68.50	92.20	91.96	25.90	22.70	8541.00	2758.81
GEN264	118.50	146.50	89.50	121.00	75.27	82.73	27.00	21.80	5778.70	2280.04
GEN266	123.00	89.50	120.00	77.50	97.55	86.44	23.35	20.75	10747.60	1738.13
GEN267	118.50	89.50	104.00	52.50	87.73	58.91	26.50	26.85	6275.00	1230.18
GEN268	106.50	112.00	97.00	93.00	91.14	83.27	26.40	23.80	8059.20	1823.26
GEN269	142.50	59.50	103.50	49.00	72.12	82.22	26.60	20.90	12059.00	858.07
GEN270	124.00	114.50	100.50	71.00	77.50	62.00	26.55	20.50	5355.00	1628.60
IR64-*Sub1*	93.00	86.50	85.50	71.00	92.01	82.17	25.80	21.60	6939.00	342.66

IR81896	100.00	191.50	90.00	148.00	89.96	77.25	26.65	20.50	4346.40	2690.46
IR84984	124.00	119.50	98.00	95.00	80.18	78.82	26.60	25.25	4519.00	1122.43
MR219	89.00	134.00	81.50	118.50	91.55	89.71	26.25	25.95	5960.00	951.32
NMR152	93.00	116.00	76.50	90.00	82.12	78.62	25.35	26.15	9756.00	2290.12
UKM5	147.00	139.50	111.50	103.00	75.89	74.26	26.20	26.05	9626.00	2605.91
$LSD_{0.05}$	62.41	78.47	55.95	73.09	32.94	32.61	3.21	7.55	6578.36	2563.25

Table S5. Principal component analysis results for NS trial of UKM5*/IR64-*Sub1* population.

Factors	PC1	PC2
Eigenvalues	1.532	1.363
Percentage of variations	23.46	18.57
Cumulative percentage	23.46	42.03
PCA variable loadings		
DTF	-0.278	0.103
PH	-0.253	-0.015
NP	-0.052	0.683
CC	0.223	-0.034
PL	0.396	-0.025
SPP	0.469	0.039
FS	0.559	0.157
SFP	0.217	0.149
TGW	-0.259	0.109
GY	-0.048	0.679

Days to flowering (DTF), plant height (PH), number of panicles (NP), chlorophyll content (CC), panicle length (PL), number of spikelet per panicle (SPP), number of filled spikelet per panicle (FS), spikelet fertility percentage (SFP), thousand-grain weight (TGW), grain yield (GY).

Table S6. Cluster means for traits evaluated in non-stress (NS) and reproductive stage drought stress (RS) trials in 2019MS.

Trial	NS			RS	
Traits	Cluster 1	Cluster 2	Cluster 3	Cluster 1	Cluster 2
DTF	0.66	0.06	-0.77	-0.20	0.19
PH	0.10	0.34	-0.71	-0.03	0.03
NP	1.06	-0.67	0.13	-0.23	0.21
CC	-0.18	-0.14	0.44	0.21	-0.20
PL	-0.46	0.06	0.36	-0.72	0.68
SPP	-0.34	-0.19	0.67	-0.72	0.67
FS	-0.51	-0.31	1.06	-0.79	0.74
SFP	-0.36	-0.16	0.64	-0.37	0.35
TGW	0.25	0.20	-0.61	-0.15	0.14
GY	1.12	-0.71	0.15	-0.36	0.34

DTF, Days to flowering; PH, plant height; NP, number of panicles; CC, chlorophyll content; PL, panicle length; SPP, number of spikelet per panicle; FS, number of filled spikelet per panicle; SFP, spikelet fertility percentage; TGW, thousand-grain weight; GY, grain yield.

Table S7. Principal component analysis results for RS trial of UKM5*/IR64-*Sub1* population.

Factor	PC1	PC2
Eigenvalues	1.725	1.267
Percentage of variations	29.74	16.04
Cumulative percentage	29.74	45.78
PCA variable loadings		
DTF	0.052	-0.351
PH	-0.016	-0.289
NP	0.112	0.318
CC	-0.138	0.043
PL	0.481	-0.225
SPP	0.487	-0.276
FS	0.549	-0.124
SFP	0.293	0.234
TGW	0.151	0.455
GY	0.294	0.535

Days to flowering (DTF), plant height (PH), number of panicles (NP), chlorophyll content (CC), panicle length (PL), number of spikelet per panicle (SPP), number of filled spikelet per panicle (FS), spikelet fertility percentage (SFP), thousand-grain weight (TGW), grain yield (GY).

Table S8. Details of marker used for genotyping.

Markers	Marker type	QTL	Category	Annealing Temperature (°C)
ART5	*Sub1C* promoter	*Sub1*	Indel	60
AEX	Functional SNP for *Sub1A*	*Sub1*	Mismatch	62
SC3	Downstream *Sub1A*	*Sub1*	SSR	55
RM416	Peak/Flanking	$qDTY_{3.1}$	SSR	55
RM520	Flanking	$qDTY_{3.1}$	SSR	55
RM28130	Peak	$qDTY_{12.1}$	SSR	55
RM511	Flanking	$qDTY_{12.1}$	SSR	55
RM1261	Flanking	$qDTY_{12.1}$	SSR	55
